# Pyloromyotomy-assisted extraction of a neglected giant duodenal spoon complicated by delayed adhesive small bowel obstruction: a case report

**DOI:** 10.1093/jscr/rjag939

**Published:** 2026-10-22

**Authors:** Amir Zamani, Seyed Mahdi Tabatabaei Naeini, Milad Dodangeh

**Affiliations:** Department of General Surgery, Loghman Hakim Hospital, Shahid Beheshti University of Medical Sciences, Tehran, Iran; Department of General Surgery, Loghman Hakim Hospital, Shahid Beheshti University of Medical Sciences, Tehran, Iran; Department of General Surgery, Loghman Hakim Hospital, Shahid Beheshti University of Medical Sciences, Tehran, Iran

**Keywords:** foreign body ingestion, duodenal foreign body, spoon ingestion, pyloromyotomy, Heineke–Mikulicz pyloroplasty, adhesive small bowel obstruction, case report

## Abstract

Intentional ingestion of long gastrointestinal foreign bodies is uncommon in adults. Giant spoon impaction in the duodenum is exceptionally rare and usually requires surgery. A 28-year-old man intentionally ingested an 18-cm metallic spoon. Computed tomography demonstrated impaction in the second part of the duodenum. After initially refusing surgery, repeat imaging suggested extra-luminal extension. Laparotomy with complete Kocher mobilization enabled extraction through a pyloromyotomy, avoiding duodenotomy, followed by Heineke–Mikulicz pyloroplasty. He was discharged on postoperative day seven. Two and a half months later, he developed adhesive small bowel obstruction requiring adhesiolysis, with an uneventful recovery. Pyloromyotomy-assisted extraction is a feasible alternative for selected giant duodenal foreign bodies when endoscopic retrieval is not possible. This case also emphasizes the need for long-term follow-up because delayed adhesive small bowel obstruction may occur after laparotomy.

## Introduction

Foreign body ingestion is a common cause of emergency department presentation, although most ingested objects pass spontaneously without intervention [1]. Approximately 10%–20% require endoscopic removal and fewer than 1% require surgery [2, 3]. The need for operative management increases when the foreign body is long, sharp, impacted, or associated with obstruction, perforation, or failed endoscopic retrieval [4].

Intentional foreign body ingestion is predominantly encountered in adults with psychiatric disorders, substance use disorders, intellectual disability, or incarceration, frequently reflecting recurrent self-harming behavior rather than accidental ingestion [5, 6]. Object length is a major determinant of spontaneous passage; items longer than ~6 cm rarely traverse the pylorus and the fixed C-loop of the duodenum, making early endoscopic removal the recommended management whenever feasible [2, 7].

Metallic spoon ingestion is exceptionally uncommon. Because of their length and rigidity, spoons rarely progress beyond the proximal gastrointestinal tract and prolonged impaction may result in pressure necrosis, perforation, fistula formation, localized inflammation, or migration into adjacent structures, substantially increasing operative complexity [3, 4, 8, 9]. Owing to the rarity of these cases, no standardized surgical approach exists. Reported techniques include gastrotomy, duodenotomy, enterotomy, and segmental bowel resection depending on the location and associated complications [5]. Although duodenotomy provides direct access, it remains technically demanding because of the retroperitoneal position of the duodenum and carries risks of leakage and delayed healing [8].

We report the successful management of a neglected giant metallic spoon impacted within the second part of the duodenum ~1 month after ingestion. The foreign body was removed through a pyloromyotomy after complete Kocher mobilization, followed by 2-layer Heineke–Mikulicz pyloroplasty. The postoperative course was later complicated by adhesive small bowel obstruction requiring re-laparotomy. This report is presented in accordance with the SCARE 2023 guidelines [10].

## Clinical timeline

The clinical timeline summarizes the patient's presentation and surgical management (Table 1 and Fig. 1).

**Table 1:** Timeline.

**Date**	**Event**
Early September 2025	Intentional ingestion of an 18-cm metallic spoon
6 October 2025	Emergency admission after acetaminophen intoxication; CT confirmed the spoon impacted in the second portion of the duodenum (D2); patient left against medical advice before definitive treatment
23 November 2025	Repeat contrast-enhanced CT showed interval migration with apparent extra-luminal extension toward the liver and right psoas region
24 November 2025	Exploratory laparotomy: Kocher mobilization, pyloromyotomy-assisted extraction, two-layer Heineke–Mikulicz pyloroplasty
28 November 2025	Follow-up CT confirmed no anastomotic/pyloroplasty leak
1 December 2025	Discharged home in good condition (postoperative day 7)
7 February 2026	Readmission with adhesive small bowel obstruction; urgent re-laparotomy with extensive adhesiolysis
1 week–6 months postoperatively	Structured outpatient follow-up; asymptomatic, normal diet, no recurrence at 1 week, 1 month, 3 months, and 6 months

**Figure 1:**
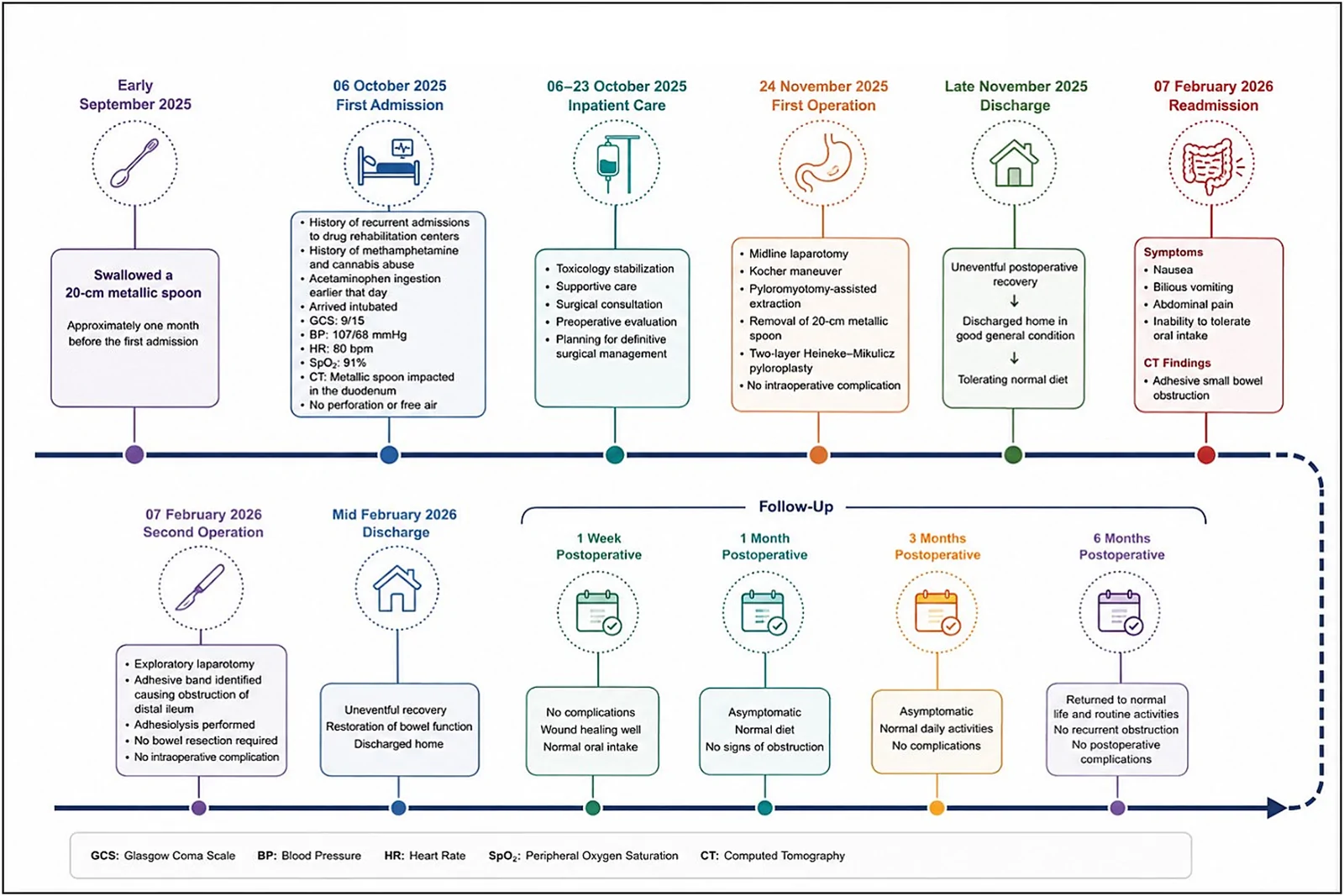
Clinical timeline illustrating the patient’s course from foreign body ingestion through delayed surgical intervention, postoperative recovery, adhesive small bowel obstruction, reoperation, and 6-month follow-up.

## Case presentation

A 28-year-old man (body mass index 22 kg/m^2^) with recurrent admissions to drug rehabilitation centers and polysubstance use, including methamphetamine and cannabis, presented on 6 October 2025, ~1 month after intentionally swallowing a metallic spoon. He had ingested acetaminophen before admission and arrived intubated for airway protection with a Glasgow Coma Scale score of 9/15. He remained hemodynamically stable (blood pressure 107/68 mmHg, heart rate 80 beats/min, oxygen saturation 91% on assisted ventilation). Laboratory investigations demonstrated mild neutrophil-predominant leukocytosis (10.7 × 10^9^/L), anemia (hemoglobin 11.7 g/dL), elevated inflammatory markers (C-reactive protein 96 mg/L; erythrocyte sedimentation rate 45 mm/h), and hypokalemia (2.8 mmol/L), while renal function, liver enzymes, coagulation profile, serum lipase, and cardiac troponin were within normal limits. Toxicology screening was positive for opiates and acetaminophen.

Non-contrast abdominal computed tomography (CT) demonstrated an ~18-cm metallic spoon impacted within the second portion of the duodenum without free intraperitoneal air (Fig. 2). Following supportive treatment and correction of electrolyte abnormalities, the patient declined definitive intervention and was discharged against medical advice.

**Figure 2:**
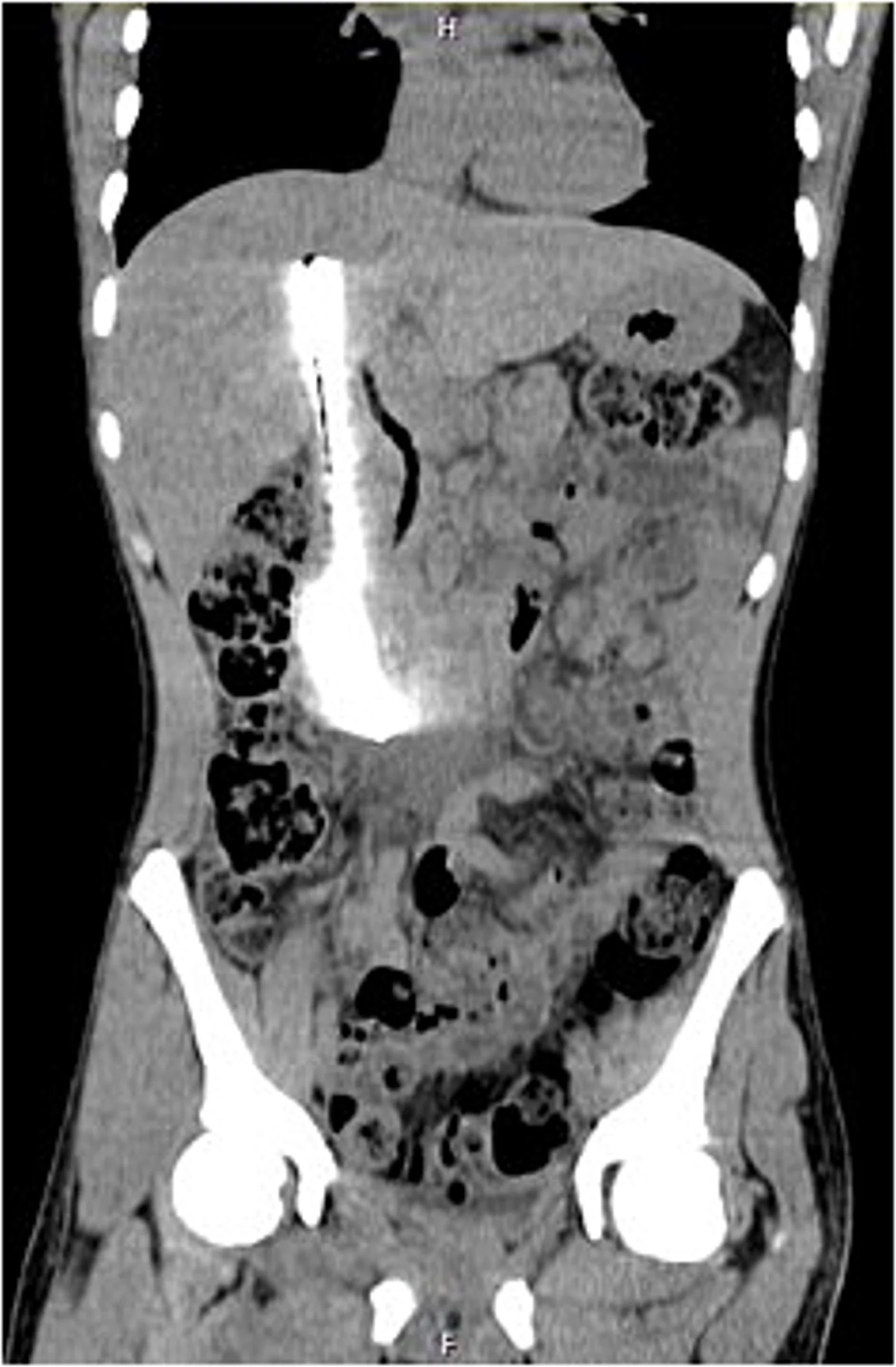
Non-contrast abdominal CT (6 October 2025) demonstrating an ~18-cm metallic spoon impacted within the second portion of the duodenum without free intraperitoneal air.

He returned ~6 weeks later for definitive treatment. Contrast-enhanced CT demonstrated persistent impaction with interval positional change and radiological suspicion of localized extra-luminal extension toward the liver and right psoas muscle (Fig. 3). Given the prolonged impaction, object size, and concern for impending perforation, primary surgical management was selected without attempted endoscopic retrieval.

**Figure 3:**
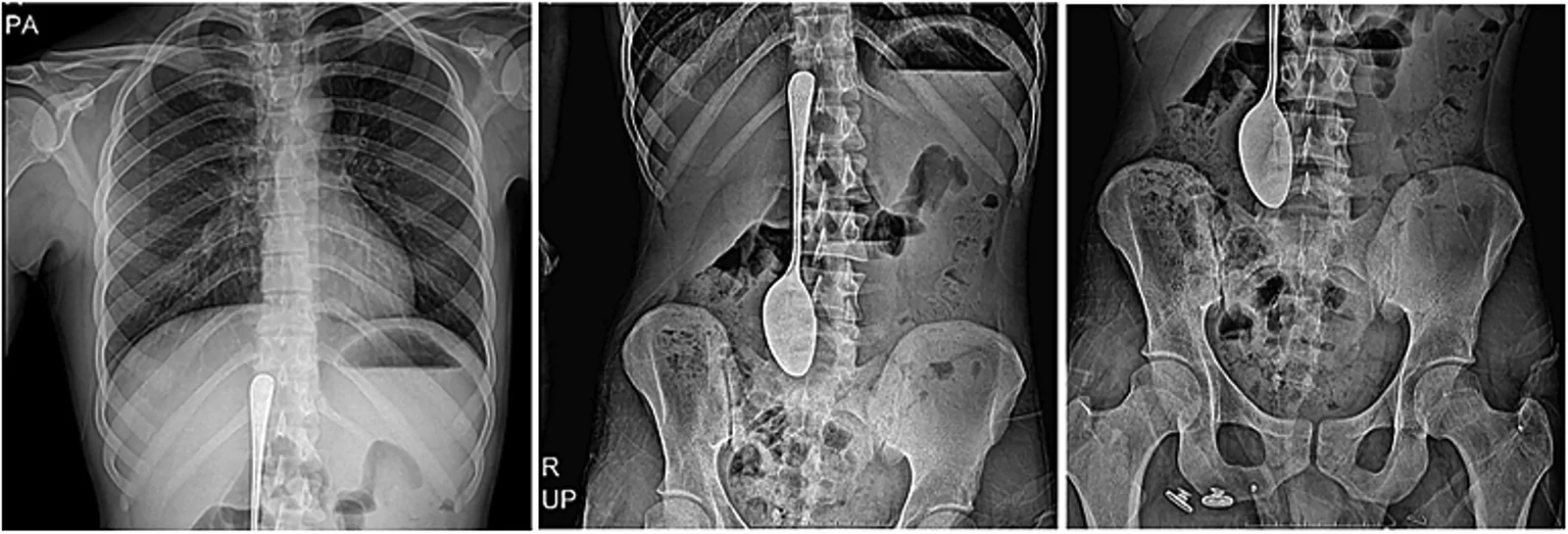
Plain abdominal radiograph (23 November 2025) demonstrating persistent impaction of the ingested spoon in the right upper abdomen, with unchanged position compared with previous imaging, raising concern for chronic impaction.

Exploratory laparotomy was performed through an upper midline incision on 24 November 2025. No free perforation, ischemia, or contamination was identified. After complete Kocher mobilization, the impacted spoon was extracted through a longitudinal pyloromyotomy, avoiding duodenotomy. The pylorus was reconstructed using a two-layer Heineke–Mikulicz pyloroplasty. Recovery was uneventful, and postoperative CT confirmed an intact repair without contrast leakage. The patient resumed oral intake gradually and was discharged on postoperative day seven.

Approximately two and a half months later, he presented with progressive abdominal pain, bilious vomiting, and intolerance of oral intake. Imaging demonstrated high-grade adhesive small bowel obstruction with dilated proximal small bowel loops and a distinct transition zone (Fig. 4). Emergency re-laparotomy revealed a dense adhesive band ~60 cm proximal to the ileocecal valve, which was successfully treated by adhesiolysis without bowel resection. Recovery was uncomplicated, and follow-up at 1 week, 1 month, 3 months, and 6 months demonstrated complete symptom resolution without recurrent obstruction or additional hospital admissions.

**Figure 4:**
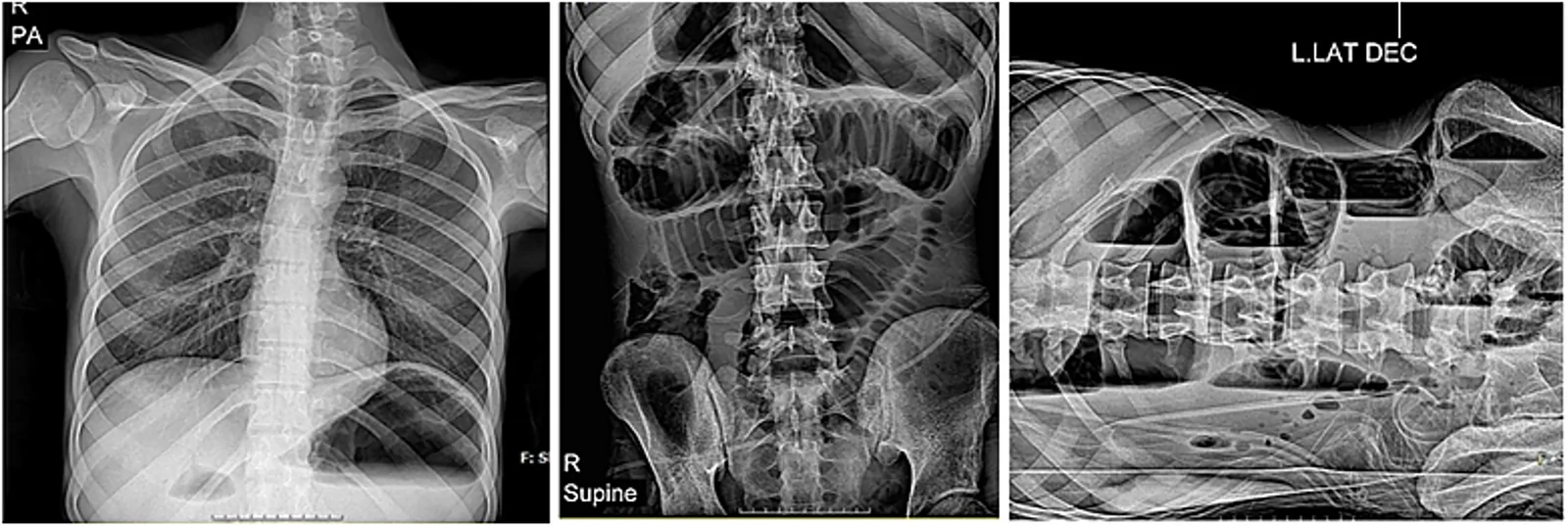
Upright and supine abdominal radiographs (7 February 2026) demonstrating multiple dilated small-bowel loops with numerous air–fluid levels, consistent with adhesive small bowel obstruction.

## Discussion

This case is notable for several uncommon features, including prolonged duodenal impaction of a giant metallic spoon, radiological suspicion of extra-luminal migration, extraction through a pyloromyotomy rather than a duodenotomy, and delayed adhesive small bowel obstruction requiring reoperation. Together, these characteristics distinguish it from most published reports, which generally describe early endoscopic retrieval or conventional duodenotomy for impacted duodenal foreign bodies [5, 8, 11].

Intentional foreign body ingestion is most frequently encountered in patients with psychiatric illness, substance use disorders, intellectual disability, or incarceration [6]. In our patient, recurrent rehabilitation admissions and methamphetamine and cannabis use were consistent with this pattern. Management should therefore include not only removal of the foreign body but also multidisciplinary evaluation to reduce the risk of recurrent ingestion. Furthermore, objects longer than 6 cm rarely traverse the pylorus and duodenum because of their anatomical configuration, making spontaneous passage of the 18-cm spoon highly unlikely and supporting current recommendations for early removal [2, 7].

Delayed presentation after ingestion of a long foreign body is uncommon but has been reported, with prolonged impaction potentially resulting in inflammation, penetration, or perforation [9]. In the present case, preoperative CT suggested partial extra-luminal extension toward the liver and right psoas muscle, substantially influencing operative planning. However, laparotomy demonstrated no gross perforation or generalized contamination. This discrepancy suggests that chronic localized inflammation may mimic extra-luminal migration on imaging or temporarily seal a microscopic perforation, emphasizing that radiological findings should always be interpreted alongside intraoperative assessment [9].

Endoscopic retrieval remains the preferred treatment for upper gastrointestinal foreign bodies whenever technically feasible [2, 7]. Nevertheless, prolonged impaction, large object size, tissue edema, and suspected extra-luminal migration markedly reduce the likelihood of successful endoscopic extraction and increase procedural risk. Recent studies recommend surgery for very large or perforating duodenal foreign bodies, supporting the operative strategy used in this patient [5].

The principal technical aspect of this case was pyloromyotomy-assisted extraction after complete Kocher mobilization. Unlike conventional duodenotomy, this approach allowed safe retrograde removal while avoiding a duodenal incision and its potential complications, including leakage and impaired healing [4, 8]. Reconstruction with a two-layer Heineke–Mikulicz pyloroplasty restored gastrointestinal continuity, and postoperative imaging confirmed the absence of leakage. Although conclusions cannot be drawn from a single case, this technique may represent a useful alternative in carefully selected patients when proximal mobilization of the foreign body is achievable [8].

The patient’s later presentation with adhesive small bowel obstruction represents a recognized consequence of laparotomy rather than the pyloromyotomy itself. Adhesive obstruction remains the most frequent long-term complication after abdominal surgery, with a reported incidence of ~4.6% [12]. Successful adhesiolysis without bowel resection and the absence of recurrent symptoms during 6 months of follow-up support the durability of the surgical reconstruction. The principal limitations of this report are its single-case design, the inability to compare surgical with endoscopic management, and the absence of intraoperative photographs, although detailed operative documentation, serial imaging, and structured follow-up strengthen the report [12].

## Conclusion

Neglected impaction of a giant metallic spoon within the duodenum is an exceptionally rare condition requiring individualized management. In patients presenting after prolonged impaction with radiological findings suggestive of extra-luminal migration, primary surgical intervention may be preferable to endoscopic retrieval. In this case, pyloromyotomy-assisted extraction following complete Kocher mobilization enabled safe removal of the foreign body while avoiding duodenotomy, and reconstruction with a two-layer Heineke–Mikulicz pyloroplasty resulted in satisfactory healing. Although the postoperative course was complicated by adhesive small bowel obstruction requiring re-laparotomy, the patient achieved complete recovery without recurrent symptoms during 6 months of follow-up. This case expands the limited literature on giant duodenal foreign bodies and suggests that pyloromyotomy-assisted extraction may represent a safe and feasible alternative in carefully selected patients when conventional endoscopic management is unsuitable.

## Data Availability

The data supporting the findings of this case report are available from the corresponding author upon reasonable request. Owing to patient confidentiality, individual-level clinical data are not publicly available.

## Patient perspective

The patient expressed satisfaction with the surgical treatment and postoperative recovery. During follow-up visits, he reported complete resolution of gastrointestinal symptoms, normal oral intake, and full return to his usual daily activities, and he expressed appreciation for the multidisciplinary care provided throughout both hospital admissions.

## References

[1] Dodangeh M, NikbakhtAmirabad O, Mahmoodi S. Intentional ingestion of a full-length table fork and multiple razor blades requiring combined enterotomy and gastrotomy: a case report. J Surg Case Rep 2026;2026:rjag445. 10.1093/jscr/rjag445 PMC13250335 42282500

[2] Ikenberry SO, Jue TL, Anderson MA et al. ASGE standards of practice committee. Management of ingested foreign bodies and food impactions. Gastrointest Endosc 2011;73:1085–91. 10.1016/j.gie.2010.11.010 21628009

[3] Velitchkov NG, Grigorov GI, Grigorov GI et al. Ingested foreign bodies of the gastrointestinal tract: retrospective analysis of 542 cases. World J Surg 1996;20:1001–5. 10.1007/s002689900152 8798356

[4] Goh BK, Chow PK, Quah HM et al. Perforation of the gastrointestinal tract secondary to ingestion of foreign bodies. World J Surg 2006;30:372–7. 10.1007/s00268-005-0490-2 16479337

[5] Ma LY, Liu ZQ, Wang Y et al. Endoscopic management of duodenal perforation caused by foreign bodies in adults: a retrospective study. United European Gastroenterol J 2024;12:390–8. 10.1002/ueg2.12505 PMC11017768 38159037

[6] King AL, Velez DR, Ahmeti M. Surgical management of an intentionally ingested vape device chronically impacted within the duodenum of an adult male. Cureus 2023;15:e39448. 10.7759/cureus.39448 PMC10289728 37362516

[7] Birk M, Bauerfeind P, Deprez PH et al. Removal of foreign bodies in the upper gastrointestinal tract in adults: European Society of Gastrointestinal Endoscopy (ESGE) clinical guideline. Endoscopy 2016;48:489–96. 10.1055/s-0042-100456 26862844

[8] Baghi I, Pirooz A, Ebrahimian R et al. Duodenal dislodgement of an ingested sharp foreign body by mucosal unfolding “Gilan maneuver”; a novel surgical approach. Int J Surg Case Rep 2022;90:106721. 10.1016/j.ijscr.2021.106721 PMC8717244 34968984

[9] Li C, Yong CC, Encarnacion DD. Duodenal perforation nine months after accidental foreign body ingestion, a case report. BMC Surg 2019;19:132. 10.1186/s12893-019-0594-5 PMC6734462 31500608

[10] Sohrabi C, Mathew G, Maria N et al. The SCARE 2023 guideline: updating consensus Surgical CAse REport (SCARE) guidelines. Int J Surg 2023;109:1136–40. 10.1097/JS9.0000000000000373 PMC10389401 37013953

[11] Eisen GM, Baron TH, Dominitz JA et al. American Society for Gastrointestinal Endoscopy. Guideline for the management of ingested foreign bodies. Gastrointest Endosc 2002;55:802–6. 10.1016/S0016-5107(02)70407-0 12024131

[12] Barmparas G, Branco BC, Schnüriger B et al. The incidence and risk factors of post-laparotomy adhesive small bowel obstruction. J Gastrointest Surg 2010;14:1619–28. 10.1007/s11605-010-1189-8 20352368

